# Associations of sex hormone ratios with metabolic syndrome and inflammation in US adult men and women

**DOI:** 10.3389/fendo.2024.1384603

**Published:** 2024-04-10

**Authors:** Pallavi Dubey, Vishwajeet Singh, Nikit Venishetty, Meesha Trivedi, Sireesha Y. Reddy, Rajkumar Lakshmanaswamy, Alok Kumar Dwivedi

**Affiliations:** ^1^ Department of Obstetrics and Gynecology, Paul L. Foster School of Medicine, Texas Tech University Health Sciences Center El Paso, El Paso, TX, United States; ^2^ Office of Research, Biostatistics and Epidemiology Consulting Lab, Texas Tech University Health Sciences Center El Paso, El Paso, TX, United States; ^3^ Department of Medical Education, Paul L. Foster School of Medicine, Texas Tech University Health Sciences Center El Paso, El Paso, TX, United States; ^4^ Department of Molecular and Translational Medicine, Paul L. Foster School of Medicine, Texas Tech University Health Sciences Center El Paso, El Paso, TX, United States; ^5^ L. Frederick Francis Graduate School of Biomedical Sciences, Texas Tech University Health Sciences Center El Paso, El Paso, TX, United States

**Keywords:** sex hormones, metabolic syndrome, C-reactive protein, cardiovascular disease, obesity, inflammation

## Abstract

**Background:**

Sex hormones play a critical role in sex differences and cardiovascular disease risk associated with metabolic syndrome (MS) and inflammation. However, the associations of sex hormone ratios with metabolic and inflammatory markers are unclear according to sex and age differences. We evaluated the associations of sex hormone ratios with MS and inflammation among males and females.

**Methods:**

A retrospective cross-sectional study was conducted by including all adults from the National Health and Nutrition Examination Survey cycles 2013-2016 and excluding any pregnant women, heart disease, diabetes, and those currently taking insulin. MS was defined using the National Cholesterol Education Program criteria and a high-sensitivity C-reactive protein (CRP) level>3 mg/L was defined as a high CRP. Measures of MS components and CRP concentrations were also analyzed. The primary exposures were testosterone to estradiol (excess androgen index), testosterone to sex hormone-binding globulin (free androgen index), and estradiol to sex hormone-binding globulin (free estradiol index). The adjusted associations were summarized with a relative risk (RR) and 95% confidence interval (CI).

**Results:**

This study included 9167 subjects with 4360 males and 4807 females. Increases in free estradiol index were positively associated with MS (RR=1.48; 95%CI: 1.39, 1.58; RR=1.31; 95%CI: 1.22, 1.40) and high CRP (RR=1.49; 95%CI: 1.25, 1.77; RR=1.26; 95%CI: 1.06, 1.50) in men with age<50 years and age≥50 years, respectively. Similarly, higher free estradiol index was also robustly associated with increased prevalence of MS (RR=1.22; 95%CI: 1.15, 1.28) and high CRP (RR=1.68; 95%CI: 1.48, 1.90) in women with age ≥50 years. Among women with age<50 years, a higher free androgen index was associated with MS (RR=1.34; 95%CI: 1.25, 1.42) and high CRP (RR=1.13; 95%CI: 1.02, 1.25). These associations were unchanged even after adjusting for all sex hormones.

**Conclusion:**

Free estradiol index was consistently and positively associated with MS and high CRP in males of all ages and older females. Free androgen index was positively associated with MS and high CRP in females with age<50 years.

## Introduction

Metabolic syndrome (MS) increases the risk of cardiovascular disease (CVD) in both sexes and is associated with multiple diseases including sleep apnea, liver disease, polycystic ovary syndrome (PCOS), and hormone-sensitive cancers (1–3). High atherosclerotic CVD accounts for 33-40% of all mortality in the United States and the European Union (4). Accumulative evidence indicates that MS-associated CVD risk is highly linked with sex and sex hormones over the lifetime (5). Although men are more likely to be at risk for CVD than women, the risk of developing CVD increases drastically after menopause in women, particularly in those with menstrual dysfunction. It has been postulated that changes in sex hormones owing to menopause transition or menstrual dysfunction may be contributory factors for adverse CVD risk in postmenopausal women (6–8). However, MS is often comorbid with systemic low-grade inflammation, making a worse prognosis (9). Emerging evidence suggests that chronic low-grade inflammation may serve as an independent marker for atherosclerotic CVD. In addition, sex-specific differences have also been observed in MS-associated inflammation (10). The current prevalence of MS is 34.7% in the United States. A notable increase in MS has become a major global health concern, especially among women, young adults, and some ethnic groups (11). Since imbalances in the sex hormones and sex chromosomes are associated with the development of metabolic abnormalities and inflammatory diseases, sex hormones may serve as global markers for MS and inflammation-associated CVD outcomes (12).

Multiple studies have investigated the association between sex hormones and MS in women with PCOS, pre- or postmenopausal women, or men (5, 13). A large meta-analysis confirmed the sex-dependent association of total testosterone (TT) and free testosterone with MS while a sex-independent association between sex hormone-binding globulin (SHBG) and MS (14). Recently, some studies highlighted a balance between TT to estradiol (E) is critical for CVD risk evaluation in men or women (15–22). Limited studies also evaluated a ratio of TT to SHBG or E to SHBG as a marker for CVD risk (23–27). However, these studies produced contradictory findings. Although several studies highlight the influence of sex hormones on inflammatory and immune cell functions, limited studies evaluated the role of a ratio of sex hormones on inflammatory markers (28–30). Several studies demonstrated that C-reactive protein (CRP) and MS are associated with carotid atherosclerosis differently in different sexes indicating different sex hormones might be associated with MS and CRP in different sexes (31). Despite the potential influence of MS and inflammation on CVD risk, the associations of ratios of sex hormones with metabolic and inflammatory markers are not evaluated according to sex and age groups. A direct comparison of the ratios of sex hormones in association with MS and inflammatory markers may provide a valuable global marker for risk stratification of MS and inflammatory-related diseases.

## Materials and methods

### Study population

This retrospective cross-sectional study was designed using data from the National Health and Nutrition Examination Survey (NHANES), a nationally representative sample of a non-institutionalized population designed to evaluate the health and nutritional status in the United States. This study contains participants from the cycle years of 2013 to 2014 and 2015-2016. The NHANES collects data after obtaining approval from the National Center for Health Statistics Research Ethics Review Board, Centers for Disease Control and Prevention, and receiving written consent from the participants. The study cohort uses a probability sampling design to represent the general US population (32). The study samples included all adults. Pregnant women, heart disease, diabetes, and currently taking insulin were excluded from the study. Of the total eligible sample (N=10,113), limited missing data on all sex hormones (N=946) were also excluded from the study.

### Exposure assessment

The serum measurements of TT (ng/dL) and E(pg/mL) were obtained using isotope dilution-liquid-chromatography tandem mass spectrometry (ID-LC-MS/MS) method while SHBG(nmol/L) concentrations were obtained using immuno-antibodies and chemo-luminescence methods. The details of quality assessment, data processing, and editing are listed in published studies (32). We computed three ratios including excess androgen index (EAI) using TT/E, free estradiol index (FEI) using E/SHBG, and free androgen index (FAI) using TT/SHBG by converting all sex hormones in the same units. The z-standardized form of these three exposures was included in the analysis. We also validated associations by categorizing sex hormones into tertiles.

### Metabolic syndrome and inflammatory markers

Trained staff collect blood samples along with laboratory and physical measures from all eligible participants using standardized protocols and methods. The MS was defined by having three of the following components: high blood pressure based on systolic blood pressure (SBP, mmHg) and diastolic blood pressure (DBP, mmHg), high triglyceride (TG, mg/dL), high fasting glucose (FG, nmol/L), increased waist circumference (WC, cm), and low high-density lipoprotein (HDL) using the National Cholesterol Education Program’s Adult Treatment Panel III guidelines (32). A high-sensitivity CRP was measured with the Beckman Coulter UniCel DxC 600 or DxC 660i Synchron Access chemistry analyzers. A CRP level >3mg/L was considered high CRP (33). We also analyzed CRP concentrations in validation analysis. Additionally, secondary outcomes in the study were the individual components of MS in addition to low-density lipoprotein (LDL). All the laboratory samples and survey data were collected at similar times in the NHANES database.

### Covariates

We retrieved data on demographic and risk factors. The demographic data included ethnicity (Hispanic, non-Hispanic), age (years), marital status (never married, married/living with partners, widowed/divorced/separated), education (<high school, high school graduate, some college degree, college graduate or above), and income in $ (<45k, 45-100k, >100k). Epidemiologic risk factors included physical status (no vs. yes), smoking status (no vs. yes), drinking status (nondrinker/occasional drinker, excessive/binge drinker, unknown), unhealthy diet (no vs. yes), body mass index (BMI-kg/m^2^) levels, and birth control pills (BCP) or hormone use (no vs. yes). A priori, all these covariates excluding BMI were adjusted in multivariable analyses (34). In addition, BCP/hormone use was also adjusted in the multivariable analysis of female samples.

### Statistical analysis

Survey weight-adjusted analysis was performed to describe and analyze data. The quantitative data were summarized with either mean and standard deviation (SD) or median with interquartile range (IQR). Categorical data were presented with frequencies and percentages. Log-transformed sex hormones and CRP concentrations were included in the analyses. All the statistical analyses were performed separately for male and female cohorts. Adjusted association of each sex hormone marker with MS was evaluated using survey-weighted Poisson regression analysis with a log link. However, the adjusted association of each sex hormone marker with each quantitative outcome was determined using survey-weighted linear regression analysis. Owing to a strong interaction between the age group (<50 vs. ≥50 years) and each sex hormone for MS and CRP outcomes, all analyses were conducted by age group (<50 vs. ≥50 years) within each sex. We further validated the associations with primary outcomes by considering the categorized form of each sex hormone using tertiles in the multivariable analyses. The results were further validated by additionally adjusting for the individual sex hormones (TT. E, and SHBG) using double-selection lasso logistic regression models. The LASSO (Least Absolute Shrinkage and Selection Operator) method under the double–selection logistic model allows us to adjust for collinear variables. Due to multiple comparisons, the primary results were considered significant at 1% alpha. The results from Poisson regression analysis were described using relative risk (RR) with 95% confidence interval (CI) whereas regression coefficient (RC) and 95%CI were used for summarizing effect sizes obtained from linear regression analyses. All statistical analyses were conducted using STATA 17 and reported using the Strengthening the Reporting of Observational Studies in Epidemiology checklist (34, 35).

## Results

A total of 9,167 subjects including 4,360 males and 4,807 females were included in the analysis. The study contains subjects with an average age (SD) of 45 (17.1) years, mostly non-Hispanics (84.7%) and married (61.3%). Relatively, male participants were younger, smokers, drinkers, physically active, and overweight or obese than females (**Table 1**). The prevalence of MS and high CRP was 42%, 25.1% in males, and 43.6% and 37.8% in females, respectively. The median E concentration was markedly lower in women with age≥50 years (22.0 vs. 241.2) while the median SHBG (48.8 vs. 31.7) was significantly higher in males with age≥50 years without much difference in TT (**Supplementary Table 1**). Most sex hormone ratios had an interaction with the age group (<50 years vs. ≥50 years) on both MS and high CRP outcomes (**Supplementary Table 2**). Accordingly, the association of sex hormones with outcomes was reported separately for each age group.

**Table 1:** Subject Characteristics, National Health and Nutrition Examination Survey (2013–2016).

Factor	Entire cohort (N=9167)	Male (N=4360)	Female (N=4807)	p-value
	N (%)	N (%)	N (%)	
**Age** (years), mean (SD)	45.15 (17.13)	43.78 (16.67)	46.42(17.45)	<0.001
Ethnicity				0.026
Non-Hispanic	6730 (84.69%)	3265 (84.09%)	3465 (85.24%)	
Hispanic	2437 (15.31%)	1095 (15.91%)	1342 (14.76%)	
Marital Status				<0.001
Never Married	1746 (18.60%)	891 (20.77%)	855 (16.59%)	
Married/Living with a partner	5168 (61.28%)	2635 (64.55%)	2533 (58.24%)	
Widowed/Divorced/Separated	1720 (16.76%)	570 (11.05%)	1150 (22.07%)	
Missing/Refused	533 (3.36%)	264 (3.63%)	269 (3.11%)	
Education				<0.001
< High School	1996 (14.54%)	1020 (15.95%)	976 (13.23%)	
High school graduate or GED	2109 (21.82%)	1062 (23.14%)	1047 (20.59%)	
Some College or AA degree	2764 (32.00%)	1193 (29.34%)	1571 (34.47%)	
College graduate or above	2294 (31.64%)	1084 (31.57%)	1210 (31.70%)	
Income ($)				<0.001
< 45k	4450 (38.81%)	2070 (36.39%)	2380 (41.06%)	
45-100k	1445 (29.67%)	1171 (30.60%)	1274 (28.80%)	
>100k	1572 (25.26%)	785 (26.66%)	787 (23.96%)	
Missing	700 (6.26%)	334 (6.34%)	366 (6.18%)	
Smoking				<0.001
No	5523 (58.62%)	2230 (52.55%)	3293 (64.26%)	
Yes	3634 (41.38%)	2125 (47.45%)	1509 (35.74%)	
Type of drinker				<0.001
Nondrinkers/occasional drinkers	1472 (12.13%)	478 (8.84%)	994 (15.18%)	
Excessive/binge drinkers	3832 (46.91%)	2255 (55.94%)	1577 (38.54%)	
Unknown	3863 (40.96%)	1627 (35.21%)	2236 (46.29%)	
Unhealthy diet				0.002
No	6390 (72.77%)	3023 (70.43%)	3367 (74.93%)	
Yes	2777 (27.23%)	1337 (29.57%)	1440 (25.07%)	
Physical Activity				<0.001
No	2712 (24.74%)	1030 (19.29%)	1682 (29.80%)	
Yes	6455 (75.26%)	3330 (80.71%)	3125 (70.20%)	
BMI (kg/m^2^)				0.001
Underweight/Normal	3010 (32.01%)	1408 (29.18%)	1602 (34.64%)	
Overweight/Obese	6075 (67.21%)	2913 (69.91%)	3162 (64.70%)	
Missing	82 (0.78%)	39 (0.91%)	43 (0.66%)	
BCP/Hormone				
No	1338 (10.88%)	NA	1338 (20.97%)	
Yes	2985 (36.56%)	NA	2985 (70.49%)	
Unknown	4844 (52.56%)	NA	484 (8.54%)	

SD, Standard deviation; BMI, Body Mass Index; BCP, Birth Control Pills; GED, Graduate Equivalent Degree; NA, Not Applicable. Data are expressed with N(%) otherwise specified.

### Associations of sex hormone ratios with MS and CRP in males by age group

**Figure 1:**
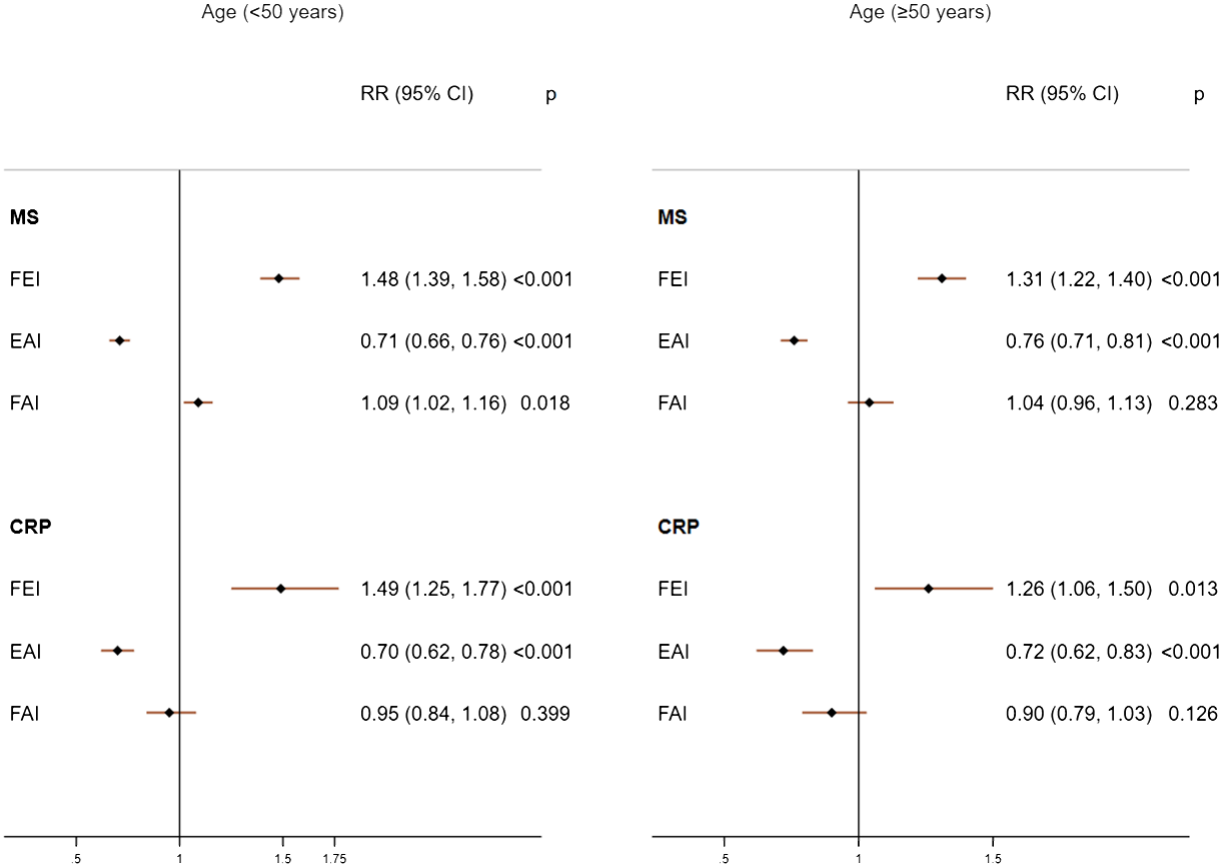
Adjusted associations of sex hormone ratios with metabolic syndrome (MS) and high C-reactive protein (CRP) in males according to age groups. FEI, Free Estradiol Index; EAI, Excess Androgen Index; FAI, Free Androgen Index; RR, Relative Risk; CI, Confidence Interval. Regression models were adjusted for age, ethnicity, marital status, education, income, physical activity, smoking, drinking, and unhealthy diet.

**Figure 2:**
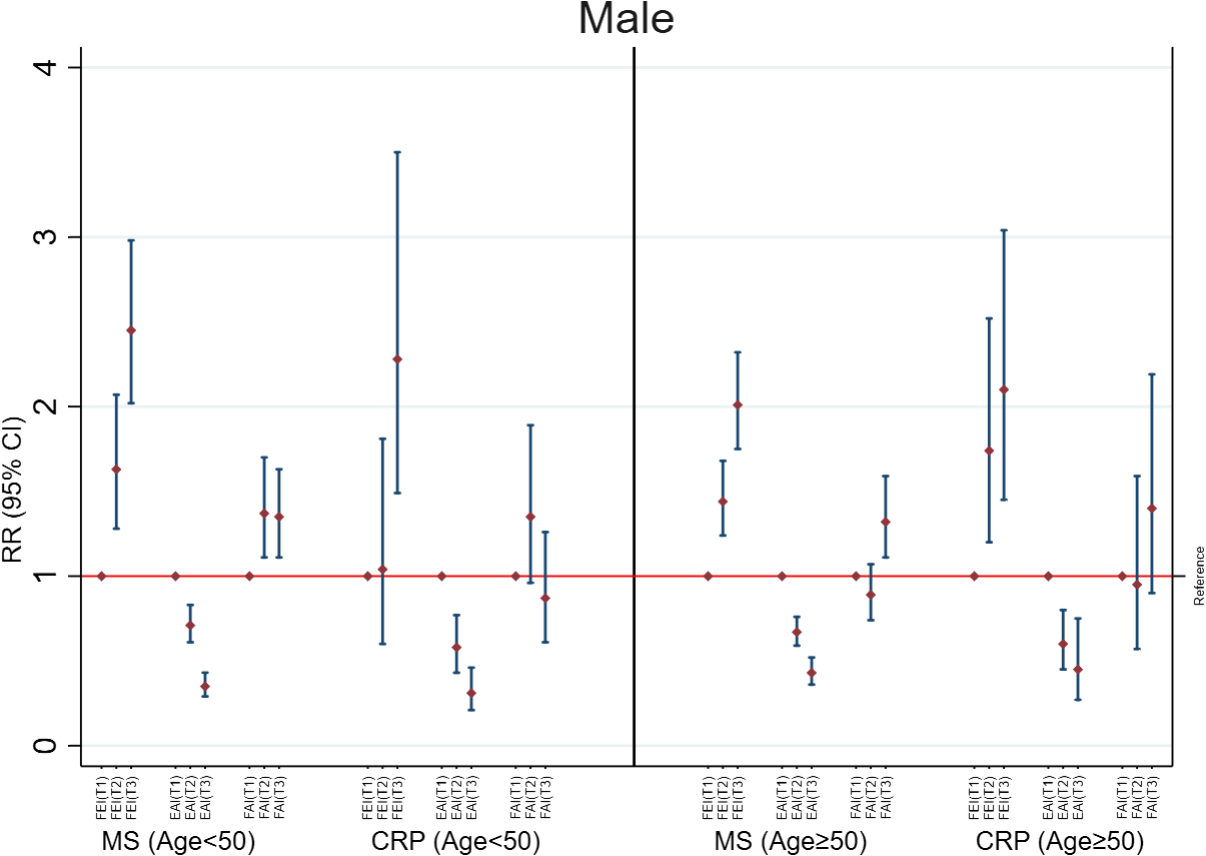
Adjusted associations of categorized sex hormone ratios with metabolic syndrome (MS) and high C-reactive protein (CRP) in males according to age groups. FEI, Free Estradiol Index; EAI, Excess Androgen Index; FAI, Free Androgen Index; RR, Relative Risk; CI, Confidence Interval. T1, First Tertile (reference category); T2, Second Tertile; T3, Third Tertile. Regression models were adjusted for age, ethnicity, marital status, education, income, physical activity, smoking, drinking, and unhealthy diet.

Increases in FEI (RR=1.48; 95%CI: 1.39, 1.58) were strongly associated with MS to a greater extent than EAI (RR=0.71; 95%CI: 0.66, 0.76) and FAI levels (RR=1.09; 95%CI: 1.02, 1.16) in males with age <50 years (**Figure 1**). The FEI remained strongly associated with MS compared to FAI or EAI even after additionally adjusting for E, TT, and SHBG concentrations (**Supplementary Table 3**). Moreover, a high FEI was markedly associated with the increased prevalence of MS compared to all sex hormones including low SHBG, TT, or high E (**Figure 2**, **Supplementary Table 4**). Similarly, increases in FEI (RR=1.49; 95%CI: 1.25, 1.77) were also associated with high CRP to a greater extent than EAI (RR=0.70; 95%CI: 0.62, 0.78) in males with age <50 years. However, the FAI was not associated with high CRP (RR= 0.95; 95%CI: 0.84, 1.08) (**Figure 1**). In all sensitivity analyses, increases in FEI remained statistically significantly associated with high CRP after additionally adjusting for E, TT, and SHBG concentrations (**Supplementary Table 3**) or CRP concentrations (**Supplementary Table 5**). A high FEI was also markedly associated with high CRP to a greater extent than other combinations of sex hormones (**Figure 2** and **Supplementary Table 4**).

Among males with age≥50 years, higher FEI levels were strongly associated with MS (RR=1.31; 95%CI: 1.22, 1.40) and high CRP (RR=1.26; 95%CI: 1.06, 1.50). Similarly, increases in EAI were also inversely associated with MS (RR=0.76; 95%CI: 0.71, 0.81) and high CRP (RR= 0.72; 95%CI: 0.62, 0.83). In contrast, FAI was not associated with MS (RR= 1.04; 95%CI: 0.96, 1.13) or high CRP (RR= 0.90; 95%CI: 0.79, 1.03) (**Figure 1**). The FEI remained the strongest factor positively associated with MS or high CRP in all sensitivity analyses including after additionally adjusting for E, TT, and SHBG concentrations (**Supplementary Table 3**) or analysis of CRP concentrations (**Supplementary Table 5**). In the categorized analysis of sex hormones, a high FEI was consistently associated with MS and high CRP than individual sex hormones or their combinations (**Figure 2**, **Supplementary Table 4**).

### Associations of sex hormone ratios with MS and CRP in females by age group

**Figure 3:**
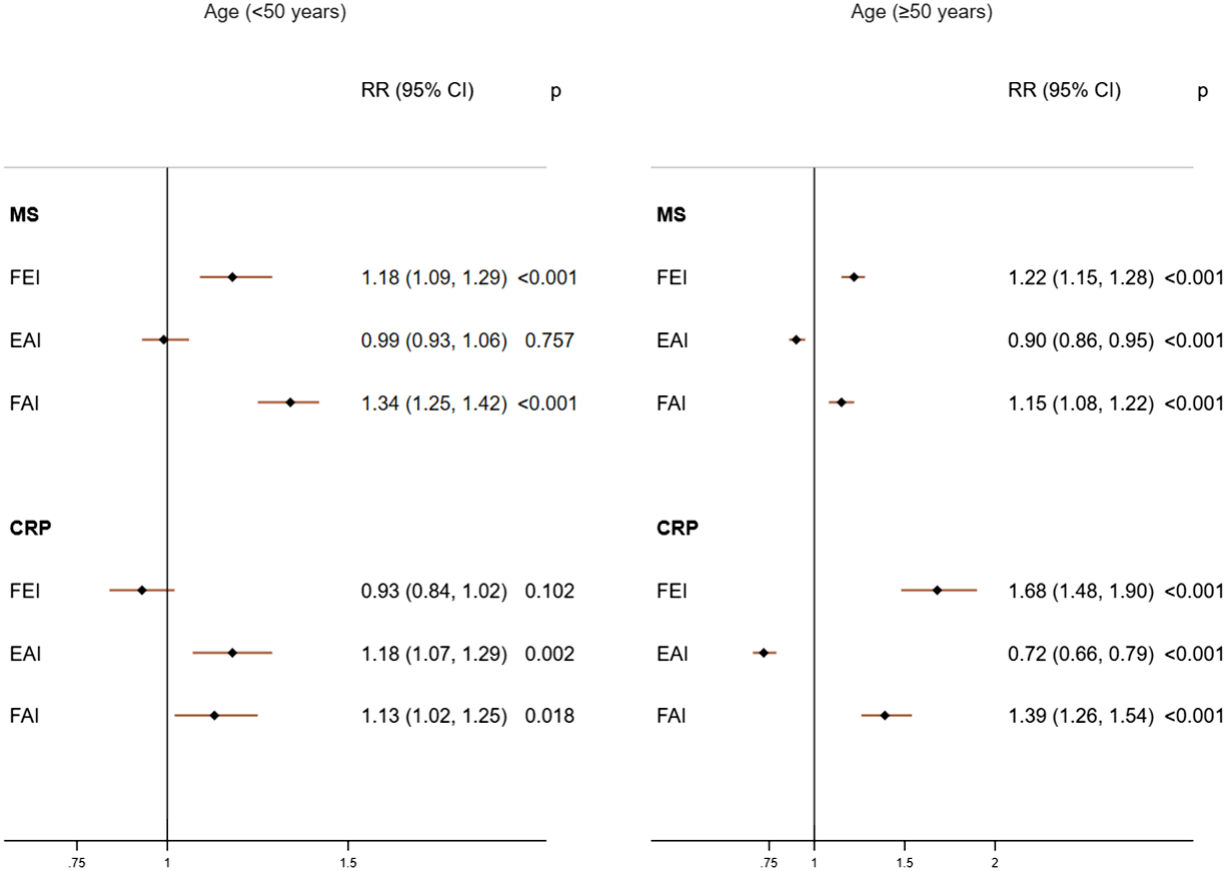
Adjusted associations of sex hormone ratios with metabolic syndrome (MS) and high C-reactive protein (CRP) in females according to age groups. FEI, Free Estradiol Index; EAI, Excess Androgen Index; FAI, Free Androgen Index; RR, Relative Risk; CI, Confidence Interval. Regression models were adjusted for age, ethnicity, marital status, education, income, physical activity, smoking, drinking, unhealthy diet, and birth control pills/hormone use.

**Figure 4:**
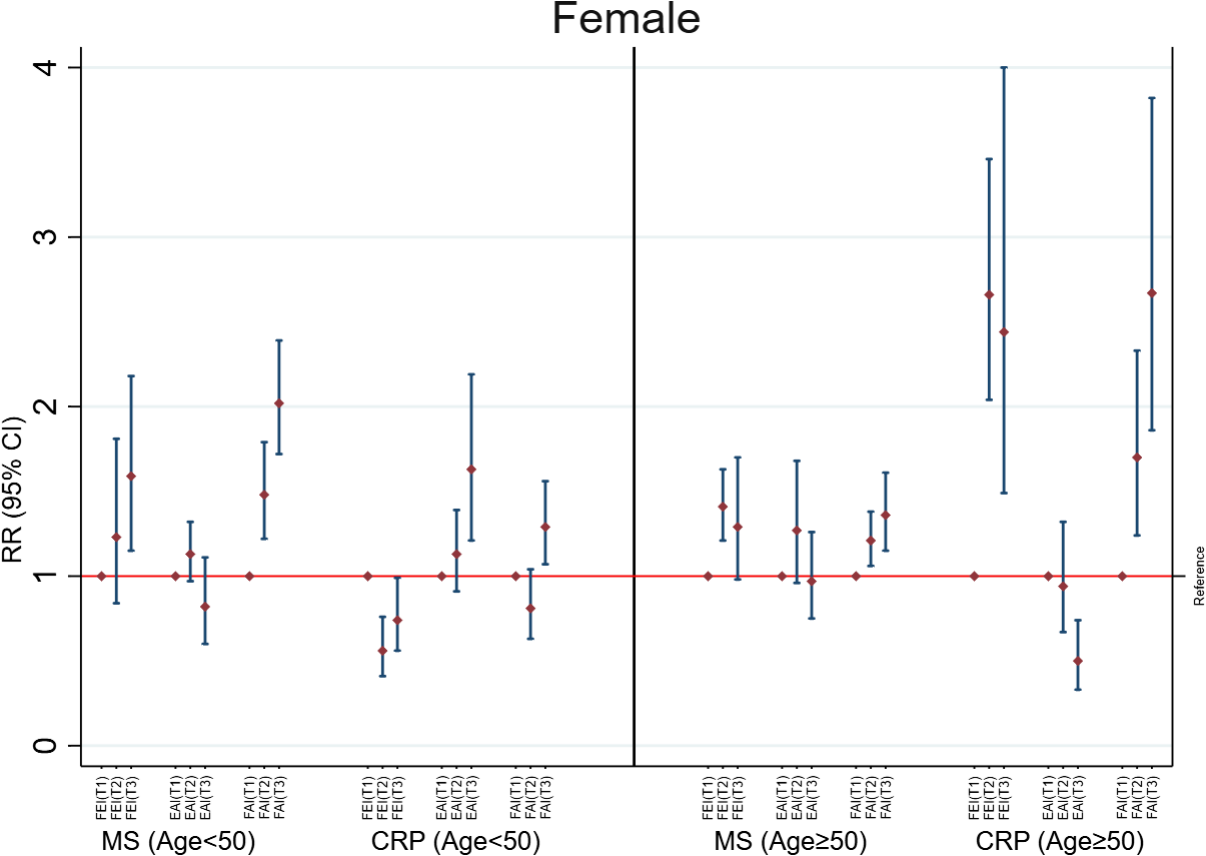
Adjusted associations of categorized sex hormone ratios with metabolic syndrome (MS) and high C-reactive protein (CRP) in females according to age groups. FEI, Free Estradiol Index; EAI, Excess Androgen Index; FAI, Free Androgen Index; RR, Relative Risk; CI, Confidence Interval. T1, First Tertile (reference category); T2, Second Tertile; T3, Third Tertile. Regression models were adjusted for age, ethnicity, marital status, education, income, physical activity, smoking, drinking, unhealthy diet, and birth control pills/hormone use.

In females of age <50 years, only higher FAI levels were consistently and positively associated with MS (RR= 1.34; 95%CI: 1.25, 1.42) and high CRP (RR=1.13; 95%CI: 1.02, 1.25) (**Figure 3**). This association was unchanged in additional adjusted analyses (**Supplementary Table 6**), categorized analysis of sex hormones or sex hormone ratios (**Figure 4**, **Supplementary Table 7**), or analysis of CRP concentrations (**Supplementary Table 8**). In females with age≥50 years, higher concentrations of FEI (RR=1.22; 95%CI: 1.15, 1.28) were associated with MS to a greater extent than FAI (RR=1.15 95%CI: 1.08, 1.22) or EAI (RR=0.90; 95%CI: 0.86, 0.95). Similarly, higher concentrations of FEI (RR= 1.68; 95%CI: 1.48, 1.90) were markedly associated with high CRP than FAI (RR= 1.39; 95%CI: 1.26, 1.54) or EAI (RR= 0.72; 95%CI: 0.66, 0.79) (**Figure 3**). These associations were also confirmed in the categorized analysis of sex hormone ratios (**Figure 4** or **Supplementary Table 7**) or additional adjusted analyses (**Supplementary Table 6**) or CRP concentration (**Supplementary Table 8**) analyses.

### Associations of sex hormone ratios with individual components of MS by sex and age group

Among males with age<50 years, both EAI and FEI were associated with all individual components of MS. However, a higher FEI (RC=26.3; 95%CI: 19.6, 33.0) was strongly associated with higher triglyceride compared to EAI (RC=-23.8; 95%CI: -30.4, -17.2). The EAI was inversely associated with waist circumference (RC=-8.6; 95%CI:-9.7, -7.6) to a greater extent than FEI (RC=7.1; 95%CI: 6.4, 7.8) (**Supplementary Table 9**). More components of MS were associated with the EAI than the FEI in males with age≥50 years. Although not statistically significant, the EAI was associated with increased LDL levels (RC=2.3; 95%CI: -0.3, 4.9). Only FAI was markedly associated with all individual components of MS except for triglyceride in females with age<50 years. However, the FEI was associated with all individual components of MS to a greater extent than EAI or FAI among females with age ≥50 years (**Supplementary Table 9**).

## Discussion

In our study, the FEI was found to be associated with MS and CRP outcomes to a greater extent than EAI or FAI in males across age groups. In addition, the FEI remained greatly associated with MS and CRP outcomes in females with age≥50 years. However, the FAI was positively associated with MS and CRP in females with age<50 years. These findings were confirmed in multiple sensitivity analyses including after additionally adjusting for each sex hormone concentration. These findings suggest a regular evaluation of sex hormone ratios, particularly FEI in both sexes and FAI in younger women. These evaluations may help physicians to stratify individuals at risk for CVD and develop potential strategies for early management of MS and CRP through maintaining sex hormone ratios.

We observed FEI as the strongest factor associated with MS and high CRP in younger or older men. To the best of our knowledge, there is no study directly evaluating the association between FEI with MS or CRP in young men. In elderly men, only one study reported associations between FEI and MS and inflammatory markers. This study on older Italian men found a strong association between free estradiol and MS supporting our study findings (36). Our findings that a higher FEI consistently associates with adverse metabolic and inflammatory profiles compared to EAI or FAI in older women have also been supported in emerging studies (24, 37, 38). Exogenous E treatment appears to be associated with the increased risk of stroke in postmenopausal women suggesting that reducing free E levels could potentially reduce the risk of MS and inflammation and subsequently CVD events (6, 24). As confirmed in multiple studies (30, 39, 40) and meta-analyses (14, 41), SHBG and TT were also inversely associated with MS and high CRP in our study. Although the association between E and MS has been conflicting among men and women (29, 36, 37, 42, 43), the FEI was positively associated with MS and high CRP even after adjusting for TT, E, and SHBG concentrations indicating the usefulness of FEI in the context of MS and CRP-related diseases.

In addition to FEI, we also observed an inverse association between EAI and MS and high CRP in men. Consistent with our findings, multiple studies reported the protective effects of a high EAI for MS and its components, inflammation, and cardiac events in men (16, 18, 19, 28). Contrary to our study findings, a few studies showed that a higher EAI is adversely associated with some metabolic abnormalities (44) and cerebrovascular disease (21) in elderly men. These contradictory associations particularly in elderly men may be due to a positive association between a higher TT and LDL cholesterol as observed in our study and other studies (45). In our healthy women sample, EAI was not consistently associated with MS and CRP outcomes. In contrast, multiple studies reported that a higher EAI is associated with incident MS (46), cardiometabolic diseases in women with menopause transition (22), development of CVD in postmenopausal women (17), and cardiometabolic markers in women with CHD (15). The role of the EAI in older men and women needs to be further explored after adjusting for SHBG and E levels.

In younger women, FAI was found to be the most critical factor for MS and CRP in our study. Hyperandrogenic profile as measured with FAI has been observed as a typical feature of MS in premenopausal women (26). A recent study further attested a link between androgenicity and the risk of developing MS before menopause using a Coronary Artery Risk Development in Young Adults (CARDIA) study (47). In addition to the FEI, we also observed a higher FAI associated with MS and CRP in women with age ≥50 years. Several studies confirmed our findings related to FAI for most metabolic and inflammatory markers in postmenopausal women (8, 23, 29, 48, 49). Rexrode et al. (25) reported a direct comparison of all combinations of sex hormones with CVD events among postmenopausal women without hormonal therapy and demonstrated that higher FAI levels are associated with CVD events. Moreover, Phillips et al. (42) showed that elevated free testosterone but not SHBG or E is associated with an increased degree of coronary artery disease. These findings confirm the potential utility of the FAI for atherosclerotic CVD risk stratification in women. In our study, the direction of associations of sex hormone ratios with MS and high CRP was generally consistent except in women of ages <50 years. A high FEI was positively associated with MS while inversely associated with high CRP in younger females. In addition, a high FAI was only associated with MS but not with high CRP in younger males while a high EAI was only associated with high CRP but not with MS in younger females. Chen et al. (31) demonstrated that CRP and MS differently predict carotid atherosclerosis in men and women. They reported CRP levels are associated with extracranial carotid artery plaques in men while MS is associated with common carotid artery intima-media thickness in women. These findings explain the potential sex hormones and sex differences related to MS and CRP-induced diseases as discussed in several studies (5, 50, 51).

Although it is unclear how a higher FEI can influence MS and CRP in men and postmenopausal women, a higher FEI above a threshold likely induces hypoandrogenemia in males and hyperandrogenism in postmenopausal females, which is associated with insulin resistance and obesity leading to MS and systematic inflammation (5, 12). Since E is primarily produced through the aromatization of TT to E in males, higher free E levels can be detrimental to cardiometabolic outcomes in aging or TT-deficient men. Owing to numerous factors, especially inflammation, insulin resistance, and obesity, the level of biologically active E (FEI) is affected in menopausal women and excess FEI can affect cardiometabolic health as confirmed in several studies (52). TT and E both are involved in oxidative phosphorylation in the mitochondria affecting adenosine triphosphate production leading to mitochondrial dysfunction, MS, and inflammation (53). Moreover, deficiency in the functions of androgen receptors and estrogen receptors has the potential to affect metabolic health. Our study also identified SHBG as a consistent marker inversely associated with MS and CRP in both sexes across age groups. In addition to FAI, lower SHBG concentrations emerged to be a robust marker for MS and CRP in our study of younger females. Since SHBG is associated with reduced E to TT ratio levels in older men (54) and TT in postmenopausal women (55), lower SHBG can influence free TT and E levels affecting cardiometabolic health in both sexes. Multiple studies confirm that SHBG has a direct effect on metabolic health and diabetes through SHBG polymorphisms and apolipoprotein B (56, 57). Recent studies discovered SHBG in the human heart produced by cardiomyocytes in dilated cardiomyopathy patients, indicating that SHBG transports sex steroids into the heart and may have a direct effect on heart condition (58). Lifestyle, dietary changes, and treatments can be used to enhance SHBG expressions and functions (59). In older subjects, estrogen blockers may be used to reduce biologically active E levels. Furthermore, prevention strategies such as minimizing environmental exposures and enhancing immunity may be universally adopted across all populations (60, 61).

### Strengths and limitations

To the best of our knowledge, this study for the first time demonstrated the potential association between FEI, MS, and CRP in males and females by age group. Unlike our study, most studies did not report a direct comparison of all sex hormone ratios in adjusted analysis or by age group. Our stringent exclusion criteria and comprehensive and sensitivity analyses attest to the reliability of our findings obtained in this study. Our study is the most comprehensive US population-based study reporting the role of different sex hormone ratios with MS, its components, and inflammatory markers separately for males and females for different age groups. Along with these strengths, our study had some limitations. Owing to cross-sectional analysis, a causal link between sex hormones and MS and inflammatory markers cannot be established with our study. Because of different timing of measurement of sex hormones, a single assessment may not be an appropriate reflection of overall exposure. Although we accounted for all available confounders in this study, some additional unmeasured covariates and hormones may affect the associations in our study. We were unable to directly compute free testosterone or estradiol levels rather indirectly estimated by combining sex hormones with SHBG.

## Conclusions

In our study, increases in FEI appeared to be strongly associated with metabolic and inflammatory markers in males across age groups. In addition, FEI was also markedly associated with a high prevalence of MS and high CRP in females of age≥ 50 years. In younger females, a higher FAI was associated with metabolic and inflammatory markers. In addition, lower SHBG concentrations were also associated with MS and high CRP in both sexes across age groups. These findings confirm the potential role of sex hormone ratios in metabolic and inflammation processes in both sexes. A regular evaluation of sex hormone ratios specific to sex is critical across age groups for CVD risk stratification and early prevention. A longitudinal study is required to validate the findings obtained in our study.

## Data availability statement

Publicly available datasets were analyzed in this study. This data can be found here: https://www.cdc.gov/nchs/nhanes/index.htm.

## Ethics statement

Ethical approval was not required for the studies involving humans because the study dataset is a publicly available database without PHI. The studies were conducted in accordance with the local legislation and institutional requirements. Written informed consent for participation was not required from the participants or the participants’ legal guardians/next of kin in accordance with the national legislation and institutional requirements because This is a publicly available database (NHANES) without PHI. The NHANES collects data after obtaining approval from the National Center for Health Statistics Research Ethics Review Board, Centers for Disease Control and Prevention, and receiving written consent from the participants.

## Author contributions

PD: Conceptualization, Data curation, Validation, Visualization, Writing – original draft, Writing – review & editing, Resources. VS: Data curation, Visualization, Writing – review & editing, Formal analysis, Methodology, Software. NV: Data curation, Visualization, Writing – review & editing, Project administration, Resources. MT: Data curation, Project administration, Resources, Visualization, Writing – review & editing. SR: Resources, Visualization, Writing – review & editing, Conceptualization, Supervision, Validation. RL: Conceptualization, Supervision, Validation, Visualization, Writing – review & editing. AD: Conceptualization, Supervision, Validation, Visualization, Writing – review & editing, Data curation, Investigation, Methodology, Project administration, Writing – original draft.

## References

[1] Dubey P Reddy SY Alvarado L Manuel SL Dwivedi AK. Prevalence of at-risk hyperandrogenism by age and race/ethnicity among females in the United States using NHANES III. Eur J Obstet Gynecol Reprod Biol. (2021) 260:189–97. doi: 10.1016/j.ejogrb.2021.03.033 33838556

[2] Mottillo S Filion KB Genest J Joseph L Pilote L Poirier P. The metabolic syndrome and cardiovascular risk a systematic review and meta-analysis. J Am Coll Cardiol. (2010) 56:1113–32. doi: 10.1016/j.jacc.2010.05.034 20863953

[3] Dwivedi AK Vishwakarma D Dubey P Reddy S. Association of polycystic ovary syndrome with cardiovascular disease among female hospitalizations in the United States. Eur J Endocrinol. (2023) 188:555–63. doi: 10.1093/ejendo/lvad067 37307574

[4] Roger VL Go AS Lloyd-Jones DM Benjamin EJ Berry JD Borden WB. Heart disease and stroke statistics–2012 update: a report from the American Heart Association. Circulation. (2012) 125:e2–20. doi: 10.1161/CIR.0b013e31823ac046 22179539 PMC4440543

[5] Faulkner JL Belin de Chantemele EJ. Sex hormones, aging and cardiometabolic syndrome. Biol Sex Differ. (2019) 10:30. doi: 10.1186/s13293-019-0246-6 31262349 PMC6604485

[6] Boardman HM Hartley L Eisinga A Main C Roque i Figuls M Bonfill Cosp X. Hormone therapy for preventing cardiovascular disease in post-menopausal women. Cochrane Database Syst Rev. (2015) 2015:CD002229. doi: 10.1002/14651858 25754617 PMC10183715

[7] Gerdts E Regitz-Zagrosek V. Sex differences in cardiometabolic disorders. Nat Med. (2019) 25:1657–66. doi: 10.1038/s41591-019-0643-8 31700185

[8] Das DV Saikia UK Sarma D. Sex hormone levels - estradiol, testosterone, and sex hormone binding globulin as a risk marker for atherosclerotic coronary artery disease in post-menopausal women. Indian J Endocrinol Metab. (2019) 23:60–6. doi: 10.4103/ijem.IJEM_505_18 PMC6446685 31016155

[9] Esser N Legrand-Poels S Piette J Scheen AJ Paquot N. Inflammation as a link between obesity, metabolic syndrome and type 2 diabetes. Diabetes Res Clin Pract. (2014) 105:141–50. doi: 10.1016/j.diabres.2014.04.006 24798950

[10] Ter Horst R van den Munckhof ICL Schraa K Aguirre-Gamboa R Jaeger M Smeekens SP. Sex-specific regulation of inflammation and metabolic syndrome in obesity. Arterioscler Thromb Vasc Biol. (2020) 40:1787–800. doi: 10.1161/ATVBAHA.120.314508 PMC7310302 32460579

[11] Hirode G Wong RJ. Trends in the prevalence of metabolic syndrome in the United States, 2011-2016. JAMA. (2020) 323:2526–8. doi: 10.1001/jama.2020.4501 PMC7312413 32573660

[12] Willemars MMA Nabben M Verdonschot JAJ Hoes MF. Evaluation of the interaction of sex hormones and cardiovascular function and health. Curr Heart Fail Rep. (2022) 19:200–12. doi: 10.1007/s11897-022-00555-0 PMC9329157 35624387

[13] Kim C Halter JB. Endogenous sex hormones, metabolic syndrome, and diabetes in men and women. Curr Cardiol Rep. (2014) 16:467. doi: 10.1007/s11886-014-0467-6 24585109 PMC4010316

[14] Brand JS van der Tweel I Grobbee DE Emmelot-Vonk MH van der Schouw YT. Testosterone, sex hormone-binding globulin and the metabolic syndrome: a systematic review and meta-analysis of observational studies. Int J Epidemiol. (2011) 40:189–207. doi: 10.1093/ije/dyq158 20870782

[15] Dai W Li Y Zheng H. Estradiol/Testosterone imbalance: impact on coronary heart disease risk factors in postmenopausal women. Cardiology. (2012) 121:249–54. doi: 10.1159/000337274 22572464

[16] Ali Hamza M Abdulhameed A Ali Mansour A. Total testosterone to estradiol ratio as a predictor marker of metabolic syndrome in males. Arch Razi Inst. (2022) 77:351–7. doi: 10.22092/ARI.2021.356607.1878 PMC9288628 35891738

[17] Zhao D Guallar E Ouyang P Subramanya V Vaidya D Ndumele CE. Endogenous sex hormones and incident cardiovascular disease in post-menopausal women. J Am Coll Cardiol. (2018) 71:2555–66. doi: 10.1016/j.jacc.2018.01.083 PMC5986086 29852978

[18] Antonio L Wu FC O’Neill TW Pye SR Carter EL Finn JD. Associations between sex steroids and the development of metabolic syndrome: a longitudinal study in European men. J Clin Endocrinol Metab. (2015) 100:1396–404. doi: 10.1210/jc.2014-4184 25636052

[19] Tivesten A Mellstrom D Jutberger H Fagerberg B Lernfelt B Orwoll E. Low serum testosterone and high serum estradiol associate with lower extremity peripheral arterial disease in elderly men. The MrOS Study in Sweden. J Am Coll Cardiol. (2007) 50:1070–6. doi: 10.1016/j.jacc.2007.04.088 17825717

[20] Awadji FB Huang B Sasmita BR Chigbo Obiegbusi S Czika A Xue Y. Association between testosterone/estradiol ratio and risk of cardiometabolic diseases in women at menopause transition age. Clin Exp Obstet Gynecol. (2022) 49:(12):260:1–9. doi: 10.31083/j.ceog4912260

[21] Gong Y Xiao H Li C Bai J Cheng X Jin M. Elevated t/e2 ratio is associated with an increased risk of cerebrovascular disease in elderly men. PloS One. (2013) 8:e61598. doi: 10.1371/journal.pone.0061598 23637864 PMC3634802

[22] Awadji FB Huang B Sasmita BR Obiegbusi SC Czika A Xue Y. Association between testosterone/estradiol ratio and risk of cardiometabolic diseases in women at menopause transition age. Clin Exp Obstet Gynecol. (2022) 49:260. doi: 10.31083/j.ceog4912260

[23] Weinberg ME Manson JE Buring JE Cook NR Seely EW Ridker PM. Low sex hormone–binding globulin is associated with the metabolic syndrome in postmenopausal women. Metabolism. (2006) 55:1473–80. doi: 10.1016/j.metabol.2006.06.017 PMC1633722 17046549

[24] Lee JS. Prospective study of endogenous circulating estradiol and risk of stroke in older women. Arch Neurol. (2010) 67:195. doi: 10.1001/archneurol.2009.322 20142527 PMC4406483

[25] Rexrode KM Manson JE Lee IM Ridker PM Sluss PM Cook NR. Sex hormone levels and risk of cardiovascular events in postmenopausal women. Circulation. (2003) 108:1688–93. doi: 10.1161/01.CIR.0000091114.36254.F3 12975257

[26] Korhonen S Hippelainen M Vanhala M Heinonen S Niskanen L. The androgenic sex hormone profile is an essential feature of metabolic syndrome in premenopausal women: a controlled community-based study. Fertil Steril. (2003) 79:1327–34. doi: 10.1016/S0015-0282(03)00347-9 12798879

[27] Sutton-Tyrrell K Wildman RP Matthews KA Chae C Lasley BL Brockwell S. Sex-hormone-binding globulin and the free androgen index are related to cardiovascular risk factors in multiethnic premenopausal and perimenopausal women enrolled in the Study of Women Across the Nation (SWAN). Circulation. (2005) 111:1242–9. doi: 10.1161/01.CIR.0000157697.54255.CE 15769764

[28] van Koeverden ID de Bakker M Haitjema S van der Laan SW de Vries JPM Hoefer IE. Testosterone to oestradiol ratio reflects systemic and plaque inflammation and predicts future cardiovascular events in men with severe atherosclerosis. Cardiovasc Res. (2019) 115:453–62. doi: 10.1093/cvr/cvy188 30052805

[29] Maggio M Ceda GP Lauretani F Bandinelli S Corsi AM Giallauria F. SHBG, sex hormones, and inflammatory markers in older women. J Clin Endocrinol Metab. (2011) 96:1053–9. doi: 10.1210/jc.2010-1902 PMC3070258 21239514

[30] Laaksonen DE Niskanen L Punnonen K Nyyssönen K Tuomainen TP Salonen R. Sex hormones, inflammation and the metabolic syndrome: a population-based study. Eur J Endocrinol. (2003) 149:601–8. doi: 10.1530/eje.0.1490601 14641004

[31] Chen PC Chien KL Hsu HC Su TC Chang CW Sung FC. C-reactive protein and the metabolic syndrome correlate differently with carotid atherosclerosis between men and women in a Taiwanese community. Metabolism. (2008) 57:1023–8. doi: 10.1016/j.metabol.2008.01.023 18640377

[32] Dubey P Reddy SY Singh V Shi T Coltharp M Clegg D. Association of exposure to phthalate metabolites with sex hormones, obesity, and metabolic syndrome in US women. JAMA Netw Open. (2022) 5:e2233088. doi: 10.1001/jamanetworkopen.2022.33088 36149653 PMC9508659

[33] Yeh ET Willerson JT. Coming of age of C-reactive protein: using inflammation markers in cardiology. Circulation. (2003) 107:370–1. doi: 10.1161/01.CIR.0000053731.05365.5A 12551854

[34] Dwivedi AK. How to write statistical analysis section in medical research. J Investig Med. (2022) 70:1759–70. doi: 10.1136/jim-2022-002479 PMC9726973 35710142

[35] Dwivedi AK Shukla R. Evidence-based statistical analysis and methods in biomedical research (SAMBR) checklists according to design features. Cancer Rep (Hoboken). (2020) 3:e1211. doi: 10.1002/cnr2.1211 32794640 PMC7941456

[36] Maggio M Lauretani F Ceda GP Bandinelli S Basaria S Paolisso G. Estradiol and metabolic syndrome in older italian men: The InCHIANTI Study. J Androl. (2010) 31:155–62. doi: 10.2164/jandrol.108.006098 PMC2842460 19059904

[37] Kalish GM Barrett-Connor E Laughlin GA Gulanski BI Postmenopausal Estrogen/Progestin Intervention T. Association of endogenous sex hormones and insulin resistance among postmenopausal women: results from the Postmenopausal Estrogen/Progestin Intervention Trial. J Clin Endocrinol Metab. (2003) 88:1646–52. doi: 10.1210/jc.2002-021375 12679451

[38] Shakir YA Samsioe G Nyberg P Lidfeldt J Nerbrand C Agardh CD. Do sex hormones influence features of the metabolic syndrome in middle-aged women? A population-based study of Swedish women: the Women’s Health in the Lund Area (WHILA) Study. Fertil Steril. (2007) 88:163–71. doi: 10.1016/j.fertnstert.2006.11.111 17383645

[39] Li C Ford ES Li B Giles WH Liu S. Association of testosterone and sex hormone-binding globulin with metabolic syndrome and insulin resistance in men. Diabetes Care. (2010) 33:1618–24. doi: 10.2337/dc09-1788 PMC2890370 20368409

[40] Bianchi VE. The anti-inflammatory effects of testosterone. J Endocr Soc. (2019) 3:91–107. doi: 10.1210/js.2018-00186 30582096 PMC6299269

[41] Brand JS Rovers MM Yeap BB Schneider HJ Tuomainen TP Haring R. Testosterone, sex hormone-binding globulin and the metabolic syndrome in men: an individual participant data meta-analysis of observational studies. PloS One. (2014) 9:e100409. doi: 10.1371/journal.pone.0100409 25019163 PMC4096400

[42] Phillips GB Pinkernell BH Jing TY. Relationship between serum sex hormones and coronary artery disease in postmenopausal women. Arterioscler Thromb Vasc Biol. (1997) 17:695–701. doi: 10.1161/01.ATV.17.4.695 9108782

[43] Cheng KH Huang SP Huang CN Lee YC Chu CS Chang CF. The impact of estradiol and 1,25(OH)2D3 on metabolic syndrome in middle-aged Taiwanese males. PloS One. (2013) 8:e60295. doi: 10.1371/journal.pone.0060295 23555948 PMC3610656

[44] Olasore HSA Oyedeji TA Olawale MO Ogundele OI Faleti JO. Relationship between testosterone-estradiol ratio and some anthropometric and metabolic parameters among Nigerian men. Metabol Open. (2023) 18:100249. doi: 10.1016/j.metop.2023.100249 37396673 PMC10313505

[45] Khaw KT Dowsett M Folkerd E Bingham S Wareham N Luben R. Endogenous testosterone and mortality due to all causes, cardiovascular disease, and cancer in men: European prospective investigation into cancer in Norfolk (EPIC-Norfolk) Prospective Population Study. Circulation. (2007) 116:2694–701. doi: 10.1161/CIRCULATIONAHA.107.719005 18040028

[46] Torrens JI Sutton-Tyrrell K Zhao X Matthews K Brockwell S Sowers M. Relative androgen excess during the menopausal transition predicts incident metabolic syndrome in midlife women: study of Women’s Health Across the Nation. Menopause. (2009) 16:257–64. doi: 10.1097/gme.0b013e318185e249 PMC2950016 18971793

[47] Vu TT Pirzada A Lewis CE Schreiner PJ Liu K Sternfeld B. Androgenicity in young women and development of metabolic syndrome before menopause: the CARDIA and CARDIA women’s studies. J Endocr Soc. (2024) 8:bvad174. doi: 10.1210/jendso/bvad174 38213908 PMC10783251

[48] Liang J Peng Q Yang X Yang C. The association between serum testosterone levels and metabolic syndrome among women. Diabetol Metab Syndr. (2021) 13:26. doi: 10.1186/s13098-021-00643-6 33676567 PMC7937283

[49] Liu C Zhao M Zhao Y Hu Y. Association between serum total testosterone levels and metabolic syndrome among adult women in the United States, NHANES 2011-2016. Front Endocrinol (Lausanne). (2023) 14:1053665. doi: 10.3389/fendo.2023.1053665 36843599 PMC9946982

[50] Poznyak AV Sukhorukov VN Guo S Postnov AY Orekhov AN. Sex differences define the vulnerability to atherosclerosis. Clin Med Insights Cardiol. (2023) 17:11795468231189044. doi: 10.1177/11795468231189044 37529084 PMC10387777

[51] Sciarra F Campolo F Franceschini E Carlomagno F Venneri MA. Gender-specific impact of sex hormones on the immune system. Int J Mol Sci. (2023) 24:1–12. doi: 10.3390/ijms24076302 PMC10094624 37047274

[52] Patel S Homaei A Raju AB Meher BR. Estrogen: The necessary evil for human health, and ways to tame it. BioMed Pharmacother. (2018) 102:403–11. doi: 10.1016/j.biopha.2018.03.078 29573619

[53] Yin L Luo M Wang R Ye J Wang X. Mitochondria in sex hormone-induced disorder of energy metabolism in males and females. Front Endocrinol (Lausanne). (2021) 12:749451. doi: 10.3389/fendo.2021.749451 34987473 PMC8721233

[54] de Ronde W van der Schouw YT Muller M Grobbee DE Gooren LJ Pols HA. Associations of sex-hormone-binding globulin (SHBG) with non-SHBG-bound levels of testosterone and estradiol in independently living men. J Clin Endocrinol Metab. (2005) 90:157–62. doi: 10.1210/jc.2004-0422 15509641

[55] Karim R Mack WJ Hodis HN Roy S Stanczyk FZ. Influence of age and obesity on serum estradiol, estrone, and sex hormone binding globulin concentrations following oral estrogen administration in postmenopausal women. J Clin Endocrinol Metab. (2009) 94:4136–43. doi: 10.1210/jc.2009-0643 PMC2775642 19808850

[56] Ding EL Song Y Manson JE Hunter DJ Lee CC Rifai N. Sex hormone–binding globulin and risk of type 2 diabetes in women and men. New Engl J Med. (2009) 361:1152–63. doi: 10.1056/NEJMoa0804381 PMC2774225 19657112

[57] Onat A Hergenc G Karabulut A Albayrak S Can G Kaya Z. Serum sex hormone-binding globulin, a determinant of cardiometabolic disorders independent of abdominal obesity and insulin resistance in elderly men and women. Metabolism. (2007) 56:1356–62. doi: 10.1016/j.metabol.2007.05.020 17884445

[58] Caldwell JD Jirikowski GF. An active role for steroid-binding globulins: an update. Horm Metab Res. (2013) 45:477–84. doi: 10.1055/s-00000025 23549676

[59] Qu M Feng C Wang X Gu Y Shang X Zhou Y. Association of serum testosterone and luteinizing hormone with blood pressure and risk of cardiovascular disease in middle-aged and elderly men. J Am Heart Assoc. (2021) 10:e019559. doi: 10.1161/JAHA.120.019559 33739129 PMC8174322

[60] Dwivedi AK Vishwakarma D Dubey P Reddy SY. Air pollution and the heart: updated evidence from meta-analysis studies. Curr Cardiol Rep. (2022) 24:1811–35. doi: 10.1007/s11886-022-01819-w 36434404

[61] Dwivedi AK Dubey P Reddy SY Clegg DJ. Associations of glycemic index and glycemic load with cardiovascular disease: updated evidence from meta-analysis and cohort studies. Curr Cardiol Rep. (2022) 24:141–61. doi: 10.1007/s11886-022-01635-2 35119682

