# Supplementary material for: Associations of sex hormone ratios with metabolic syndrome and inflammation in US adult men and women
Source: Front Endocrinol (Lausanne). 2024 Apr 10;15:1384603. doi: 10.3389/fendo.2024.1384603 (PMC11039964; doi:10.3389/fendo.2024.1384603)
Supplement: Supplementary file 1 [file Table_1.docx]

**Supplementary Tables**

**Supplementary Table S1. Distribution of outcome measures and exposures in males and females by age group**

|  | **Male** | | | **Female** | | |
| --- | --- | --- | --- | --- | --- | --- |
|  | **Age <50 years** | **Age ≥50 years** | **p-value** | **Age <50 years** | **Age ≥50 years** | **p-value** |
|  | **Mean (SD)** | **Mean (SD)** |  | **Mean (SD)** | **Mean (SD)** |  |
| **Outcome measures** |  |  |  |  |  |  |
| MS, N (%) | 851 (34.46%) | 921 (54.98%) | <0.001 | 980 (33.37%) | 1229 (57.22%) | <0.001 |
| CRP (>3), N (%) | 298 (23.83%) | 263 (27.16%) | 0.369 | 509 (37.33%) | 405 (38.36%) | 0.764 |
| CRP^*^ (mg/L) | 1.20 (0.50, 2.90) | 1.60 (0.80, 3.30) | 0.002^#^ | 1.90 (0.60, 5.10) | 2.10 (1.00, 4.70) | 0.014^#^ |
| FG (nmol/L) | 5.60 (0.87) | 5.94 (1.12) | <0.001 | 5.33 (0.83) | 5.74 (0.97) | <0.001 |
| TG^*^ (mg/dL) | 97 (65, 153) | 97 (65, 143) | 0.395^#^ | 74 (54, 108) | 98 (68, 145) | <0.001^#^ |
| HDL (mg/dL) | 47.53 (12.97) | 51.55 (17.40) | <0.001 | 57.74 (16.49) | 64.40 (19.53) | <0.001 |
| LDL (mg/dL) | 114.98 (33.85) | 115.84 (35.92) | 0.719 | 105.12 (32.39) | 120.79 (35.70) | <0.001 |
| SBP (mmHg) | 120.02 (12.04) | 128.79 (17.72) | <0.001 | 113.34 (12.61) | 128.77 (18.83) | <0.001 |
| DBP (mmHg) | 71.13 (10.79) | 72.23 (11.60) | 0.048 | 68.9 (10.05) | 69.58 (10.66) | 0.072 |
| WC (cm) | 98.23 (15.85) | 103.60 (14.14) | <0.001 | 94.35 (17.47) | 98.83 (15.13) | <0.001 |
| **Exposures** |  |  |  |  |  |  |
| FEI ^*^ | 0.28 (0.19, 0.40) | 0.18 (0.12, 0.25) | <0.001^#^ | 0.41 (0.19, 0.75) | 0.04 (0.02, 0.09) | <0.001^#^ |
| EAI^*^ | 169.77 (127.66, 221.67) | 155.59 (121.08, 204.01) | <0.001^#^ | 3.44 (1.73, 7.67) | 27.43 (15.30, 43.68) | <0.001^#^ |
| FAI^*^ | 45.33 (35.17, 57.66) | 28.53 (22.32, 35.73) | <0.001^#^ | 1.21 (0.73, 2.03) | 0.90 (0.55, 1.44) | <0.001^#^ |
| TT^*^ (nmol/L) | 14.23 (10.69, 18.39) | 13.71 (10.48, 17.91) | 0.031^#^ | 0.79 (0.57, 1.10) | 0.60 (0.40, 0.87) | <0.001^#^ |
| SHBG^*^ (nmol/L) | 31.69 (22.52, 42.12) | 48.75 (36.02, 65.86) | <0.001^#^ | 62.34 (41.40, 99.28) | 66.07 (45.97, 93.42) | 0.399^#^ |
| E^*^ (pmol/L) | 84.99 (67.18, 106.10) | 89.21 (70.49, 108.30) | 0.114^#^ | 241.20 (108.67, 469.93) | 22.03 (11.86, 42.95) | <0.001^#^ |

Abbreviations: SD, Standard Deviation; TT, Total Testosterone; SHBG, Sex Hormone-Binding Globulin; E, Estradiol, FEI, Free Estradiol Index computed using E to SHBG ratio; EAI, Excess Androgen Index computed using TT to E ratio; FAI, Free Androgen Index computed using TT to SHBG ratio; FI, Fasting Insulin; FG, Fasting Glucose; TG, Triglyceride; TC, Total Cholesterol; HDL, High-Density Lipoproteins; LDL, Low-Density Lipoproteins; SBP, Systolic Blood Pressure; DBP, Diastolic Blood Pressure; WC, Waist Circumference; BMI, Body Mass Index; CRP, C - Reactive Protein; WBC, White Blood Cell; MS, Metabolic Syndrome.

*Data are expressed with median with interquartile range (IQR); #log values were compared.

**Supplementary Table S2. Significant interactions of age group (<50 vs. ≥50 years) with sex hormone ratios for MS and high CRP outcomes in males and females**

|  | **Male** | **Female** |
| --- | --- | --- |
|  | **p-value** | **p-value** |
| **Metabolic syndrome** |  |  |
| FEI | 0.046 | 0.151 |
| EAI | 0.083 | 0.182 |
| FAI | 0.120 | <0.001 |
| **High C-Reactive Protein** |  |  |
| FEI | 0.251 | <0.001 |
| EAI | 0.499 | <0.001 |
| FAI | 0.906 | 0.051 |

Abbreviations: TT, Total Testosterone; SHBG, Sex Hormone-Binding Globulin; E, Estradiol, FEI, Free Estradiol Index computed using E to SHBG ratio; EAI, Excess Androgen Index computed using TT to E ratio; FAI, Free Androgen Index computed using TT to SHBG ratio; Log values of sex hormone ratios were included in the regression analyses.

**Supplementary Table S3. Associations of sex hormone ratios with MS and high CRP in males by age group after additionally adjusting for total testosterone, estradiol, and sex hormone-binding globulin concentrations**

|  | **Age (<50 years)** | | **Age (≥50 years)** | |
| --- | --- | --- | --- | --- |
|  | **RR (95% CI)** | **p-value** | **RR (95% CI)** | **p-value** |
| **Metabolic syndrome** |  |  |  |  |
| FEI | 1.82 (1.48, 2.23) | <0.001 | 2.15 (1.72, 2.67) | <0.001 |
| EAI | 0.54 (0.44, 0.66) | <0.001 | 0.55 (0.43, 0.70) | <0.001 |
| FAI | 0.57 (0.48, 0.69) | <0.001 | 0.51 (0.39, 0.67) | <0.001 |
| **High C-Reactive Protein** |  | |  | |
| FEI | 2.58 (1.83, 3.63) | <0.001 | 1.89 (1.34, 2.66) | <0.001 |
| EAI | 0.45 (0.32, 0.63) | <0.001 | 0.45 (0.32, 0.63) | <0.001 |
| FAI | 0.49 (0.36, 0.66) | <0.001 | 0.58 (0.43, 0.77) | <0.001 |

Abbreviations: TT, Total Testosterone; SHBG, Sex Hormone-Binding Globulin; E, Estradiol; FEI, Free Estradiol Index computed using E to SHBG ratio; EAI, Excess Androgen Index computed using TT to E ratio; FAI, Free Androgen Index computed using TT to SHBG ratio; RR, Relative Risk; CI, Confidence Interval; CRP, C - Reactive Protein; MS, Metabolic Syndrome.

Regression models were adjusted for total testosterone, estradiol, and sex hormone-binding globulin concentrations in addition to age, ethnicity, marital status, education, income, physical activity, smoking, drinking, and unhealthy diet. Z-standardized values of the log of sex hormones were included in the double-selection lasso logistic regression.

**Supplementary Table S4. Associations of categorized sex hormones with MS and high CRP in males by age group**

|  | **Age (<50 years)** | | **Age (≥50 years)** | |
| --- | --- | --- | --- | --- |
|  | **RR (95% CI)** | **p-value** | **RR (95% CI)** | **p-value** |
| **Metabolic syndrome** |  |  |  |  |
| **TT** |  |  |  |  |
| T1 | 1 [Reference] |  | 1 [Reference] |  |
| T2 | 0.70 (0.61, 0.80) | <0.001 | 0.73 (0.65, 0.83) | <0.001 |
| T3 | 0.42 (0.34, 0.51) | <0.001 | 0.60 (0.51, 0.71) | <0.001 |
| **SHBG** |  |  |  |  |
| T1 | 1 [Reference] |  | 1 [Reference] |  |
| T2 | 0.59 (0.50, 0.69) | <0.001 | 0.87 (0.73, 1.03) | 0.097 |
| T3 | 0.35 (0.27, 0.47) | <0.001 | 0.57 (0.49, 0.67) | <0.001 |
| **E** |  |  |  |  |
| T1 | 1 [Reference] |  | 1 [Reference] |  |
| T2 | 1.14 (0.95, 1.36) | 0.147 | 1.20 (1.04, 1.39) | 0.016 |
| T3 | 1.23 (1.04, 1.45) | 0.016 | 1.21 (1.02, 1.44) | 0.032 |
| **FEI** |  |  |  |  |
| T1 | 1 [Reference] |  | 1 [Reference] |  |
| T2 | 1.63 (1.28, 2.07) | <0.001 | 1.44 (1.24, 1.68) | <0.001 |
| T3 | 2.45 (2.02, 2.98) | <0.001 | 2.01 (1.75, 2.32) | <0.001 |
| **EAI** |  |  |  |  |
| T1 | 1 [Reference] |  | 1 [Reference] |  |
| T2 | 0.71 (0.61, 0.83) | <0.001 | 0.67 (0.59, 0.76) | <0.001 |
| T3 | 0.35 (0.29, 0.43) | <0.001 | 0.43 (0.36, 0.52) | <0.001 |
| **FAI** |  |  |  |  |
| T1 | 1 [Reference] |  | 1 [Reference] |  |
| T2 | 1.37 (1.11, 1.70) | 0.005 | 0.89 (0.74, 1.07) | 0.192 |
| T3 | 1.35 (1.11, 1.63) | 0.003 | 1.32 (1.11, 1.59) | 0.004 |
| **High C-Reactive Protein** |  | |  | |
| **TT** |  |  |  |  |
| T1 | 1 [Reference] |  | 1 [Reference] |  |
| T2 | 0.56 (0.37, 0.85) | 0.009 | 0.66 (0.45, 0.96) | 0.031 |
| T3 | 0.42 (0.27, 0.64) | 0.001 | 0.56 (0.42, 0.76) | 0.001 |
| **SHBG** |  |  |  |  |
| T1 | 1 [Reference] |  | 1 [Reference] |  |
| T2 | 0.68 (0.47, 0.98) | 0.038 | 0.69 (0.46, 1.05) | 0.08 |
| T3 | 0.48 (0.31, 0.75) | 0.003 | 0.45 (0.32, 0.63) | <0.001 |
| **E** |  |  |  |  |
| T1 | 1 [Reference] |  | 1 [Reference] |  |
| T2 | 1.08 (0.74, 1.58) | 0.669 | 1.16 (0.83, 1.62) | 0.356 |
| T3 | 1.66 (1.15, 2.40) | 0.010 | 1.13 (0.76, 1.68) | 0.522 |
| **FEI** |  |  |  |  |
| T1 | 1 [Reference] |  | 1 [Reference] |  |
| T2 | 1.04 (0.6, 1.81) | 0.876 | 1.74 (1.20, 2.52) | 0.006 |
| T3 | 2.28 (1.49, 3.5) | 0.001 | 2.10 (1.45, 3.04) | 0.001 |
| **EAI** |  |  |  |  |
| T1 | 1 [Reference] |  | 1 [Reference] |  |
| T2 | 0.58 (0.43, 0.77) | 0.001 | 0.60 (0.45, 0.80) | 0.002 |
| T3 | 0.31 (0.21, 0.46) | <0.001 | 0.45 (0.27, 0.75) | 0.005 |
| **FAI** |  |  |  |  |
| T1 | 1 [Reference] |  | 1 [Reference] |  |
| T2 | 1.35 (0.96, 1.89) | 0.08 | 0.95 (0.57, 1.59) | 0.845 |
| T3 | 0.87 (0.61, 1.26) | 0.437 | 1.40 (0.90, 2.19) | 0.125 |

Abbreviations: TT, Total Testosterone; SHBG, Sex Hormone-Binding Globulin; E, Estradiol; FEI, Free Estradiol Index computed using E to SHBG ratio; EAI, Excess Androgen Index computed using TT to E ratio; FAI, Free Androgen Index computed using TT to SHBG ratio; RR, Relative Risk; CI, Confidence Interval; CRP, C - Reactive Protein; MS, Metabolic Syndrome. T1, First Tertile (reference category); T2, Second Tertile; T3, Third Tertile

Regression models were adjusted for age, ethnicity, marital status, education, income, physical activity, smoking, drinking, and unhealthy diet. Tertile values of sex hormones were included in the regression analyses.

**Supplementary Table S5. Associations of sex hormone ratios with CRP concentrations in males by age group**

|  | **Age (<50 years)** | | **Age (≥50 years)** | |
| --- | --- | --- | --- | --- |
|  | **RC (95% CI)** | **p-value** | **RC (95% CI)** | **p-value** |
| FEI | 0.37 (0.24, 0.49) | <0.001 | 0.21 (0.07, 0.36) | 0.007 |
| EAI | -0.49 (-0.57, -0.42) | <0.001 | -0.38 (-0.48, -0.27) | <0.001 |
| FAI | 0 (-0.12, 0.12) | 0.992 | -0.14 (-0.23, -0.04) | 0.010 |

Abbreviations: TT, Total Testosterone; SHBG, Sex Hormone-Binding Globulin; E, Estradiol; FEI, Free Estradiol Index computed using E to SHBG ratio; EAI, Excess Androgen Index computed using TT to E ratio; FAI, Free Androgen Index computed using TT to SHBG ratio; RC, Regression Coefficient; CI, Confidence Interval; CRP, C - Reactive Protein.

Regression models were adjusted for age, ethnicity, marital status, education, income, physical activity, smoking, drinking, and unhealthy diet. Z-standardized values of the log of sex hormone ratios were included in the regression analysis. Log-transformed values of CRP were used in the regression analyses.

**Supplementary Table S6. Associations of sex hormone ratios with MS and high CRP in females by age group after additionally adjusting for total testosterone, estradiol, and sex hormone-binding globulin concentrations**

|  | **Age (<50 years)** | | **Age (≥50 years)** | |
| --- | --- | --- | --- | --- |
|  | **RR (95% CI)** | **p-value** | **RR (95% CI)** | **p-value** |
| **Metabolic syndrome** |  |  |  |  |
| FEI | 1.03 (0.90, 1.17) | 0.700 | 1.21 (1.04, 1.40) | 0.011 |
| EAI | 0.80 (0.64, 1.00) | 0.054 | 1.01 (0.85, 1.20) | 0.925 |
| FAI | 2.31 (2.03, 2.62) | <0.001* | 1.01 (0.88, 1.15) | 0.925 |
| **High C-Reactive Protein** |  | |  | |
| FEI | 0.64 (0.54, 0.76) | <0.001 | 1.98 (1.58, 2.48) | <0.001 |
| EAI | 1.53 (1.11, 2.11) | 0.009 | 0.79 (0.60, 1.04) | 0.094 |
| FAI | 1.34 (1.08, 1.66) | 0.009 | 0.83 (0.67, 1.03) | 0.094 |

Abbreviations: TT, Total Testosterone; SHBG, Sex Hormone-Binding Globulin; E, Estradiol; FEI, Free Estradiol Index computed using E to SHBG ratio; EAI, Excess Androgen Index computed using TT to E ratio; FAI, Free Androgen Index computed using TT to SHBG ratio; RR, Relative Risk; CI, Confidence Interval; CRP, C - Reactive Protein; MS, Metabolic Syndrome.

Regression models were adjusted for total testosterone, estradiol, and sex hormone-binding globulin concentrations in addition to age, ethnicity, marital status, education, income, physical activity, smoking, drinking, unhealthy diet, and birth control pills/hormonal use. *additionally adjusted for total testosterone and estradiol concentrations, Z-standardized values of the log of sex hormone ratios were included in the double-selection lasso logistic regression.

**Supplementary Table S7. Associations of categorized sex hormones with MS and high CRP in females by age group**

|  | **Age (<50 years)** | | **Age (≥50 years)** | |
| --- | --- | --- | --- | --- |
|  | **RR (95% CI)** | **p-value** | **RR (95% CI)** | **p-value** |
| **Metabolic syndrome** |  |  |  |  |
| **TT** |  |  |  |  |
| T1 | 1 [Reference] |  | 1 [Reference] |  |
| T2 | 0.92 (0.78, 1.08) | 0.277 | 0.91 (0.81, 1.02) | 0.094 |
| T3 | 0.72 (0.60, 0.86) | 0.001 | 0.86 (0.76, 0.98) | 0.028 |
| **SHBG** |  |  |  |  |
| T1 | 1 [Reference] |  | 1 [Reference] |  |
| T2 | 0.59 (0.51, 0.69) | <0.001 | 0.80 (0.71, 0.89) | <0.001 |
| T3 | 0.39 (0.32, 0.47) | <0.001 | 0.52 (0.44, 0.61) | <0.001 |
| **E** |  |  |  |  |
| T1 | 1 [Reference] |  | 1 [Reference] |  |
| T2 | 1.34 (0.97, 1.86) | 0.076 | 1.22 (1.08, 1.38) | 0.002 |
| T3 | 1.07 (0.80, 1.42) | 0.655 | 0.96 (0.71, 1.30) | 0.790 |
| **FEI** |  |  |  |  |
| T1 | 1 [Reference] |  | 1 [Reference] |  |
| T2 | 1.23 (0.84, 1.81) | 0.274 | 1.41 (1.21, 1.63) | <0.001 |
| T3 | 1.59 (1.15, 2.18) | 0.006 | 1.29 (0.98, 1.70) | 0.070 |
| **EAI** |  |  |  |  |
| T1 | 1 [Reference] |  | 1 [Reference] |  |
| T2 | 1.13 (0.97, 1.32) | 0.124 | 1.27 (0.96, 1.68) | 0.095 |
| T3 | 0.82 (0.60, 1.11) | 0.186 | 0.97 (0.75, 1.26) | 0.831 |
| **FAI** |  |  |  |  |
| T1 |  |  |  |  |
| T2 | 1.48 (1.22, 1.79) | <0.001 | 1.21 (1.06, 1.38) | 0.006 |
| T3 | 2.02 (1.72, 2.39) | <0.001 | 1.36 (1.15, 1.61) | 0.001 |
| **High C-Reactive Protein** |  | |  | |
| **TT** |  |  |  |  |
| T1 | 1 [Reference] |  | 1 [Reference] |  |
| T2 | 1.18 (0.93, 1.50) | 0.159 | 1.21 (0.98, 1.51) | 0.075 |
| T3 | 0.91 (0.69, 1.21) | 0.509 | 1.13 (0.84, 1.52) | 0.395 |
| **SHBG** |  |  |  |  |
| T1 | 1 [Reference] |  | 1 [Reference] |  |
| T2 | 0.59 (0.47, 0.74) | <0.001 | 0.66 (0.52, 0.82) | 0.001 |
| T3 | 0.68 (0.57, 0.81) | <0.001 | 0.37 (0.24, 0.58) | <0.001 |
| **E** |  |  |  |  |
| T1 | 1 [Reference] |  | 1 [Reference] |  |
| T2 | 0.75 (0.60, 0.95) | 0.018 | 1.84 (1.39, 2.42) | <0.001 |
| T3 | 0.59 (0.46, 0.75) | <0.001 | 1.85 (1.15, 2.97) | 0.014 |
| **FEI** |  |  |  |  |
| T1 | 1 [Reference] |  | 1 [Reference] |  |
| T2 | 0.56 (0.41, 0.76) | 0.001 | 2.66 (2.04, 3.46) | <0.001 |
| T3 | 0.74 (0.56, 0.99) | 0.045 | 2.44 (1.49, 4.00) | 0.002 |
| **EAI** |  |  |  |  |
| T1 | 1 [Reference] |  | 1 [Reference] |  |
| T2 | 1.13 (0.91, 1.39) | 0.244 | 0.94 (0.67, 1.32) | 0.694 |
| T3 | 1.63 (1.21, 2.19) | 0.003 | 0.50 (0.33, 0.74) | 0.002 |
| **FAI** |  |  |  |  |
| T1 | 1 [Reference] |  | 1 [Reference] |  |
| T2 | 0.81 (0.63, 1.04) | 0.088 | 1.70 (1.24, 2.33) | 0.003 |
| T3 | 1.29 (1.07, 1.56) | 0.012 | 2.67 (1.86, 3.82) | <0.001 |

Abbreviations: TT, Total Testosterone; SHBG, Sex Hormone-Binding Globulin; E, Estradiol; FEI, Free Estradiol Index computed using E to SHBG ratio; EAI, Excess Androgen Index computed using TT to E ratio; FAI, Free Androgen Index computed using TT to SHBG ratio; RR, Relative Risk; CI, Confidence Interval; CRP, C - Reactive Protein; MS, Metabolic Syndrome. T1, First Tertile (reference category); T2, Second Tertile; T3, Third Tertile.

Regression models were adjusted for age, ethnicity, marital status, education, income, physical activity, smoking, drinking, unhealthy diet, and birth control pills/hormone use. Tertile values of sex hormones were included in the regression analyses.

**Supplementary Table S8. Associations of sex hormone ratios with CRP concentrations in females by age group**

|  | **Age (<50 years)** | | **Age (≥50 years)** | |
| --- | --- | --- | --- | --- |
|  | **RC (95% CI)** | **p-value** | **RC (95% CI)** | **p-value** |
| FEI | -0.10 (-0.23, 0.03) | 0.116 | 0.48 (0.38, 0.57) | <0.001 |
| EAI | 0.24 (0.11, 0.37) | 0.001 | -0.34 (-0.44, -0.24) | <0.001 |
| FAI | 0.21 (0.10, 0.31) | 0.001 | 0.27 (0.16, 0.38) | <0.001 |

Abbreviations: TT, Total Testosterone; SHBG, Sex Hormone-Binding Globulin; E, Estradiol; FEI, Free Estradiol Index computed using E to SHBG ratio; EAI, Excess Androgen Index computed using TT to E ratio; FAI, Free Androgen Index computed using TT to SHBG ratio; RC, Regression Coefficient; CI, Confidence Interval; CRP, C - Reactive Protein.

Regression models were adjusted for age, ethnicity, marital status, education, income, physical activity, smoking, drinking, unhealthy diet, and birth control pills/hormone use. Z-standardized values of the log of sex hormone ratios were included in the regression analysis. Log-transformed values of CRP were used in the regression analyses.

**Supplementary Table S9. Adjusted associations of sex hormone ratios with components of metabolic syndrome in males and females by age group**

|  | **Male (Age <50 years)**  **(N=2648)** | | **Male (Age ≥50 years)**  **(N=1712)** | | **Female^#^ (Age <50 years)**  **(N=2812)** | | **Female^#^ (Age ≥50 years)**  **(N=1995)** | |
| --- | --- | --- | --- | --- | --- | --- | --- | --- |
|  | **RC (95% CI)** | **p-value** | **RC (95% CI)** | **p-value** | **RC (95% CI)** | **p-value** | **RC (95% CI)** | **p-value** |
| **FEI** | | | | | | | | |
| FG | 0.13 (0.06, 0.19) | <0.001 | 0.06 (-0.02, 0.15) | 0.126 | 0.05 (0.01, 0.08) | 0.015 | 0.15 (0.09, 0.21) | <0.001 |
| HDL | -3.43 (-4.15, -2.71) | <0.001 | -4.36 (-5.56, -3.17) | <0.001 | -1.92 (-2.62, -1.22) | <0.001 | -5.07 (-6.58, -3.56) | <0.001 |
| SBP | 2.72 (1.99, 3.45) | <0.001 | 1.04 (-0.29, 2.37) | 0.121 | -0.07 (-0.71, 0.58) | 0.83 | 2.23 (1.25, 3.21) | <0.001 |
| DBP | 2.15 (1.75, 2.55) | <0.001 | 0.98 (0.23, 1.74) | 0.013 | -0.01 (-0.49, 0.47) | 0.963 | -0.26 (-0.92, 0.41) | 0.435 |
| Waist | 7.07 (6.39, 7.75) | <0.001 | 4.74 (3.63, 5.86) | <0.001 | 1.86 (1.12, 2.6) | <0.001 | 6.39 (4.96, 7.83) | <0.001 |
| TG | 26.3 (19.63, 32.97) | <0.001 | 20.85 (11.18, 30.52) | <0.001 | -2.66 (-7.23, 1.91) | 0.244 | 10.12 (4.14, 16.1) | 0.002 |
| LDL | 3.47 (0.89, 6.04) | 0.010 | -1.54 (-4.66, 1.58) | 0.321 | -1.56 (-3.69, 0.58) | 0.147 | 1.23 (-2.24, 4.7) | 0.474 |
| **EAI** | | | | | | | | |
| FG | -0.14 (-0.21, -0.07) | 0.001 | -0.14 (-0.28, 0) | 0.056 | 0 (-0.04, 0.04) | 0.998 | -0.08 (-0.15, -0.02) | 0.011 |
| HDL | 3.18 (2.41, 3.95) | <0.001 | 4.04 (3.04, 5.05) | <0.001 | 0.26 (-0.38, 0.89) | 0.414 | 3.24 (1.82, 4.66) | <0.001 |
| SBP | -3.14 (-3.82, -2.45) | <0.001 | -1.91 (-3.19, -0.62) | 0.005 | 0.77 (0.11, 1.42) | 0.023 | -1.39 (-2.33, -0.45) | 0.005 |
| DBP | -1.81 (-2.23, -1.38) | <0.001 | -0.64 (-1.27, 0) | 0.050 | 0.45 (0.06, 0.84) | 0.025 | 0.21 (-0.4, 0.81) | 0.492 |
| Waist | -8.62 (-9.65, -7.59) | <0.001 | -7.08 (-8.33, -5.82) | <0.001 | 0.83 (0.17, 1.5) | 0.015 | -4 (-5.23, -2.77) | <0.001 |
| TG | -23.78 (-30.37, -17.2) | <0.001 | -15.87 (-24.77, -6.96) | 0.001 | 4.65 (0.71, 8.6) | 0.022 | -6.22 (-11.01, -1.44) | 0.013 |
| LDL | -3.19 (-6.25, -0.13) | 0.042 | 2.29 (-0.27, 4.85) | 0.078 | 2.73 (0.77, 4.7) | 0.008 | 2.38 (-1.19, 5.94) | 0.183 |
| **FAI** | | | | | | | | |
| FG | 0.02 (-0.07, 0.1) | 0.705 | 0 (-0.08, 0.08) | 0.968 | 0.12 (0.06, 0.18) | <0.001 | 0.12 (0.05, 0.19) | 0.001 |
| HDL | -1.49 (-2.15, -0.83) | <0.001 | -1.15 (-2.42, 0.11) | 0.072 | -3.7 (-4.33, -3.06) | <0.001 | -3.13 (-4.41, -1.86) | <0.001 |
| SBP | 0.47 (-0.32, 1.26) | 0.236 | -0.3 (-1.39, 0.79) | 0.574 | 1.19 (0.58, 1.8) | <0.001 | 1.23 (0.42, 2.03) | 0.004 |
| DBP | 1.01 (0.56, 1.46) | <0.001 | 0.76 (-0.12, 1.64) | 0.089 | 0.74 (0.2, 1.28) | 0.009 | -0.06 (-0.68, 0.57) | 0.855 |
| Waist | 0.57 (-0.18, 1.33) | 0.131 | -1.06 (-2.34, 0.22) | 0.102 | 5.48 (4.6, 6.37) | <0.001 | 4.18 (3.1, 5.26) | <0.001 |
| TG | 13.26 (5.82, 20.7) | 0.001 | 10.44 (-0.29, 21.17) | 0.056 | 0.61 (-4.23, 5.45) | 0.799 | 7.2 (1.98, 12.43) | 0.009 |
| LDL | 1.39 (-2.47, 5.24) | 0.469 | 1.99 (-1.36, 5.33) | 0.235 | 1.99 (-0.06, 4.05) | 0.057 | 5.5 (2.42, 8.57) | 0.001 |

Abbreviations: TT, Total Testosterone; SHBG, Sex Hormone-Binding Globulin; E, Estradiol; FEI, Free Estradiol Index computed using E to SHBG ratio; EAI, Excess Androgen Index computed using TT to E ratio; FAI, Free Androgen Index computed using TT to SHBG ratio; RC, Regression Coefficient; CI, Confidence Interval; CRP, C - Reactive Protein.

Regression models were adjusted for age, ethnicity, marital status, education, income, physical activity, smoking, drinking, unhealthy diet, and/or birth control pills /hormone use (if female). Z-standardized values of the log of sex hormone ratios were included in the regression analyses.
